# The Relative Importance of Age at Peak Height Velocity and Fat Mass Index in High-Intensity Interval Training Effect on Cardiorespiratory Fitness in Adolescents: A Randomized Controlled Trial

**DOI:** 10.3390/children9101554

**Published:** 2022-10-13

**Authors:** Jarosław Domaradzki, Dawid Koźlenia, Marek Popowczak

**Affiliations:** 1 Unit of Biostructure, Faculty of Physical Education and Sport, Wroclaw University of Health and Sport Sciences, al. I.J. Paderewskiego 35, 51-612 Wroclaw, Poland; 2 Unit of Team Sports Games, Faculty of Physical Education and Sport, Wroclaw University of Health and Sport Sciences, al. I.J. Paderewskiego 35, 51-612 Wroclaw, Poland

**Keywords:** puberty, biological age, APHV, fat mass index, cardiorespiratory fitness, high-intensity interval training, adolescents

## Abstract

Background: This study aimed to evaluate the role of biological age in the relationship between preintervention fat mass and cardiorespiratory fitness effects (CRF) after High-Intensity Interval Training (HIIT) intervention in adolescents. Methods: A total of 141 boys and girls (16 years) were examined as control (CG) and experimental (EG) groups that conducted a 10-week HIIT implemented in physical education. Measurements: body height, body weight, fat mass index (FMI), CRF (Harvard Step Test). Predicted age at peak height velocity (APHV) was calculated. Pathway analyses were conducted to identify the influences of the measured variables. Results: APHV harmed the level of ΔFI (fitness index) (*β* = −0.270; *p* = 0.035), while HIIT had a positive effect on changes in ΔFI (*β* = 0.246; *p* = 0.002). Sex affected the changes in CRF (*β* = 0.344; *p* = 0.011). Two models were designed respective to sex. The effect of the APHV on ΔFI in girls was close to significant (*p* = 0.053) but was non-significant in boys (*p* = 0.311). Changes in CRF in girls were positively and close to significance (*p* < 0.100) affected by FMI_baseline_. Conclusion: HIIT is an effective way of exerting positive changes in CRF in adolescents, which is greater in boys than girls. APHV plays a role only in girls. The HIIT should be tailored to girls depending on their maturity status.

## 1. Introduction

Physical fitness is a powerful indicator of health in children and adolescents [1,2]. It is a construct that contains primary health-related components usually separated into cardiorespiratory fitness (CRF) and muscular fitness [3]. The primary aim of physical education is to develop physical and cardiorespiratory fitness [4]. Each physical education (PE) lesson should increase physical activity and promote activity and exercises [5]. CRF is defined as the human body’s circulatory system’s ability to supply fuel and oxygen during sustained physical activity [6]. Thus, it reflects the cardiorespiratory system’s efficiency in transporting and utilizing O_2_ through blood, using the aerobic metabolic pathways. Determining the level of the CRF has scientific and clinical applications [7]. Cardiorespiratory fitness can be measured in different ways. Field tests are usually used to estimate VO_2max_, e.g., shuttle run tests [8] or 6 min walking tests [9]. But for in-school conditions, the Harvard Step Test can be used during physical education (PE) lessons too [10]. Then it is possible to calculate the physical fitness index (FI) that defines CRF [1,11]. In the Harvard Step Test, oxygen consumption is usually estimated from equations. The validity of the Harvard Step Test to predict VO_2max_ has been proven many times [11,12,13]. Cardiorespiratory fitness is influenced by different intrapersonal factors, e.g., sex and age (both calendar and biological), and depends on body composition [14].

A relationship links CRF and body fat mass from a public health perspective. Chin et al. [15], in a study of overweight and obese adult men, noted that there were positive changes in cardiorespiratory fitness and body fat mass after implementing programs based on intense exercise. The prevalence of obesity covers every fourth child and youth in the Western world [16]. Simultaneously, most individuals with elevated fat tissue have a low level of CRF, which has declined over the past six decades [17,18,19]. In addition, when examining adult men, Murawska-Cialowicz et al. [20] showed an improvement in aerobic capacity, participants’ CRF, and a fat reduction after the 8-week High-Intensity Interval Training(HIIT) with the Tabata Protocol program. 

The relationship between cardiorespiratory fitness and age (both chronological and biological) regarding sex has been studied many times [21,22,23,24,25]. Results most often showed sex differences in relationships between age and CRF level. Cardiorespiratory fitness was significantly higher in younger girls compared to older girls and in early puberty compared to late puberty. In males, cardiorespiratory fitness was higher in younger adolescents, but no differences were observed when it was analyzed according to sexual maturation status. For the boys, the highest increase occurred when they were at an age of peak height velocity [26].

There are solid multidimensional associations between a high level of obesity, a low level of CRF, and a lack of physical activity in children. Global increment in inactivity (called “sedentary style of life”) is reflected in numbers. About 80% of young people do not perform the minimum physical activity level recommended by the World Health Organization [27,28]. Therefore, there is an urgent need to promote a physically active lifestyle. Physical activity is an effective way of regulating body mass, preventing obesity, and improving physical efficiency [29,30]. CRF can be increased by regular exercise, which is extremely important for health and quality of life [31]. A high level of CRF is associated with a higher capacity for physical activity. It is carried out more often in one’s free time or with a targeted approach during PE that ensures the appropriate intensity. This is important for maintaining an average body mass index (BMI), appropriate body fat percentage, and reducing the risk of metabolic, cardiovascular, and psychiatric diseases. In contrast, a low level of CRF is associated with insufficient physical activity, overweight and obesity, poor diet, metabolic and cardiovascular disease risk, mental health problems, and increased morbidity [32,33]. 

The relevant setting to introduce physical activity seems to be physical education lessons [9]. High-intensity exercise in physical education (PE) lessons positively correlates with improving body mass index [34,35]. Therefore, high-intensity interval training (HIIT) with a short intervention time seems to be the most appropriate method of increasing exercise intensity in PE lessons. This saves time, improves maximum oxygen uptake, and affects cardiovascular parameters, mass body composition, and CRF in adolescence [36,37]. The differences were observed in the effects of sex and BMI [38], suggesting various effects of HIIT due to internal factors in adolescents. The positive impact of the participation of pubertal children in HIIT was presented by Alvarez et al. [38] and Eddolls et al. [39].

There is a predominance in the literature of studies examining the direct effects of independent variables on CRF or BFM, very often separately and usually in simple comparisons (t-test, ANOVA, or non-parametric substitutes) and dependence analyses (using regressions correlations). The abovementioned variables indicated high coefficients of associations [34,35,36,37,40,41,42,43]. However, most determinants and correlations may act additively, strengthening or weakening their actions. Because of the interactions, these factors and variables may also indirectly influence CRF or BFM. The relationship between BFM and CRF and the effectiveness of HIIT in improving the status in both parameters has drawn interest from public health educators, PE teachers, and scientists. Despite this, investigations with more complex study designs remain scarce. The role of sex, calendar age, and different interventions is well-documented [19]. However, further research is needed to understand better the influence of the biological age on associations between CRF and body fat mass (BFM), taking HIIT effectiveness into account. Therefore, the study’s objective was to evaluate the role of biological age in associations between preintervention fat mass index status (BFM concerning body height) and the volume of changes in CRF after HIIT intervention regarding the effect of sex. It was done by designing the explanatory model of the direct and indirect paths between these variables. In the first step, the model was constructed for all individuals, both males and females. In the case of the sex moderation role, two other models (for boys and girls, separately) should be investigated. HIIT is a time-efficient training program that could be efficiently adopted into a 45 min physical education lesson. The short duration of a single lesson demands short and intense efforts, increasing the general intensity of PE and, therefore, positively influencing the cardiorespiratory and physical fitness and body composition [10,31]. Therefore, HIIT seems to be perfectly fitted into school settings. However, it is necessary to verify its effectiveness.

## 2. Materials and Methods

### 2.1. The Sampling Plan

This study was a part of the project “Physical activity and nutritional education in preventing civilization diseases—theoretical aspects and practical implications for the secondary school physical education program,” which was carried out in one of the secondary schools in Wroclaw (a city in the Lower Silesia region, Poland). The intervention program introduced into PE lessons was HIIT. In addition, students participated in additional lectures on proper nutrition. Detailed methodology descriptions were published elsewhere [10,35,36,37]. However, the role of the biological age in the relationship between preintervention FMI status (body fat mass concerning body height) and HIIT effects in CRF in adolescents was not analyzed.

### 2.2. Power Calculation

The G*Power tool (Sydenham Institute of Management Studies and Research and Entrepreneurship Education) was used to calculate the sample size [44]. Based on 80% total power, 0.05–0.01-point minimum effect size, and an *α* level of 0.05, it was calculated that 81–159 participants would be required in order to conduct analyses.

### 2.3. Participants

Initially, the study sample consisted of 187 people divided into 6 classes: 3 randomly assigned to the experimental group and 3 to the control group. Students in each class were on the same education level with the same PE education program. Before or during the intervention, 46 students were excluded (due to medical contraindications, participation in additional sports activities in the past six months, or absence from physical education lessons). Finally, 52 boys and 89 girls completed the study following their aim. Before starting the school year, experimental groups (EG) and control groups (CG) were randomly selected from all first-year students in the secondary school. There were 42 girls and 31 boys in the experimental group, while 47 girls and 21 boys were in the control group.

In the present study, concerning the aim of work, only the results of individuals who positively responded to the HIIT intervention program (for whom positive outcomes were noted) were examined. The numbers of individuals with positive changes per outcome are presented in Table 1.

### 2.4. Anthropometric Measurements

Body height (BH) measurement was performed twice with an accuracy of 0.1 cm using anthropometers (GPM Anthropological Instruments). Bodyweight (BW) and body fat mass (BFM) were measured by the InBody bioimpedance analyzer—InBody230 (InBody Co. Ltd., Cerritos, CA, USA). To calculate fat mass index (FMI), a formula similar to BMI was used: FMI (kg/m^2^) = BFM (kg)/BH (m^2^).

### 2.5. Intervention Program

Students from the experimental group (EG) performed a 14 min HIIT program introduced to a traditional PE lesson described elsewhere [10]. Briefly, here are the main assumptions. In the beginning, students performed a standardized warm-up. Directly after, they performed a 14 min HIIT during e sessions of 4 min each, with a 1 min break between sessions. The program consisted of eight cycles of two exercises with a maximum intensity exercise lasting 20 s with as many repetitions as possible. The exercises were presented on a TV screen. Cooling down was performed after the main sessions. HIIT was applied for 10 weeks of the school year. The intervention was introduced into one PE lesson weekly in the standard school curriculum between 9:00 am and 12:30 pm. Other lessons were on schedule. The procedure verifying the HIIT intensity was presented by Domaradzki et al. [10]. 

All participants were instructed to avoid extensive physical effort before the measurements [45]. During the other two physical education classes in the week, activities occurred according to the physical education program. 

### 2.6. Cardiorespiratory Fitness

The Harvard step test was used to establish the CRF, which was determined by the fitness index (FI) [1,11]. The validity of the Harvard step test to predict VO_2max_ and estimation of oxygen consumption was proved [11,12,13]. Correlations between direct and predicted VO_2max_ were calculated as r = 0:618–0.805, and the repeatability was acceptable (ICC = 0.63) [13]. According to the Harvard step test presented by Domaradzki et al. [10], participants stepped up and down on a 16.25-inch (41.3 cm) high stool at 30 cycles per minute. The exercise continued for up to 300 s. Each student could finish the test early if he or she were tired. Recovery pulse was recorded within 1.5 min of recovery. Polar H1 heart rate monitors (Polar, Polar Electro; Kempele, Finland) were used to measure the heart rate of each student before, during, and after the test according to the procedure outlined by Domaradzki et al. [10] The Fitness Index was calculated using the following formula [46]: FI = (100 × L)/(5.5 × *p*), where L is the duration of the test in seconds, L < 300 s and *p* is the heart rate within 1.5 min after the subject stopped the test.

### 2.7. Prediction Age at Peak Height Velocity (APHV)

The variation in the tempo of biological growth with the indication of the moment in maturity for both sexes in EG and CG was assessed using formulas proposed by Moore et al. [47]. Age at peak height velocity (APHV) was predicted using sex-specific regression equations: 

girls—maturity offset = −7.709133 + (0.0042232 × (age × BH));

boys—maturity offset = −7.999994 + (0.0036124 × (age × BH)).

Age at peak height velocity (APHV) was calculated as calendar age–maturity offset (MO) for all individuals.

### 2.8. Theoretical Model

The hypothetical explanatory model was constructed using path analysis [48,49]. GLS (generalized) evaluated the interrelations between sex, APHV, HIIT intervention, and BFM on CRF changes. 

**Figure 1:**
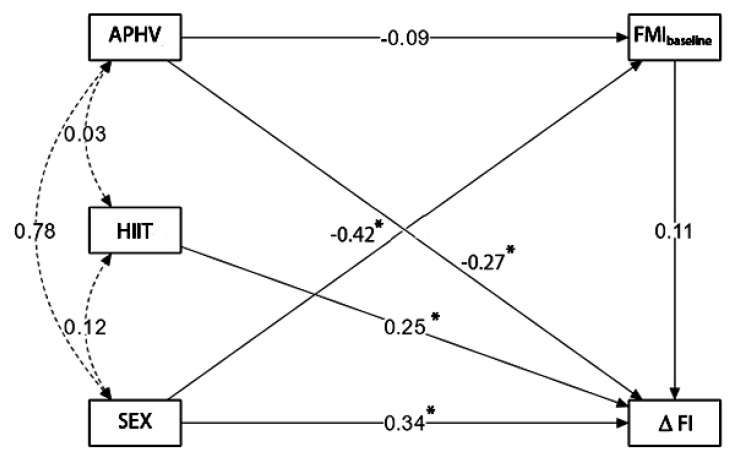
The path model, constructed for all participants (including boys and girls), shows the association between changes in FI and APHV, HIIT intervention, and sex as exogenous variables with the preintervention FMI. Abbreviations: APHV—age at peak height velocity, HIIT—HIIT groups (EG-1 coded, CG-0 coded), SEX—males (1 coded) and females (0 coded), FMI_baseline_—fat mass index preintervention, ΔFI—changes in fitness index, *—statistically significant path.

The primary outcomes (endogenous variables) were measurements of the BFM preintervention and differences between the post-, and preintervention fitness index (FI) received from results of the Harvard Step Test. Changes in FI (reflected CRF level) mirrored CRF gain after HIIT intervention. The explanatory (exogenous) variables were sex, APHV, and HIIT. The tested hypotheses were: Sex, APHV, and HIIT directly affect BFM and the volume of CRF changes. Sex and APHV have an indirect effect on CRF, mediated by BFM. Potential sex moderation was assessed, including all participants in one model. A statistically significant moderation role of the sex contributed to conducting analyses for boys and girls separately. The results are presented in Figure 1, where rectangles represent variables and arrows—associations (paths) from the independent to the dependent [50]. 

### 2.9. Statistical Analysis

Outcomes were preintervention FMI values and Δ (post-pre changes) of the FI. The normality of data distribution was assessed using the Shapiro–Wilk test. Descriptive statistics of continuous variables (APHV, MO, body height, body weight, fat mass index, and physical index) were calculated as means, standard deviations, and 95% confidence intervals (CI). ANOVA evaluated differences between 4 groups: EG boys, CG boys, EG girls, CG girls in APHV, MO, preintervention FMI, and changes in FI (ΔFI). The F-values and *p*-values were calculated.

The path analysis was conducted to assess direct and indirect effects. Standardized coefficients were calculated to quantify those effects. The maximum likelihood (ML) method, implemented in Jamovi software (Sydney, Australia), was used. Algorithms implemented in the Advanced Path Models 1.0.4 module were performed (Jamovie, v.1.6, 2020, The Jamovi Project, Sydney, Australia). Model suitability was determined based on *β*^2^ (*p* > 0.05 suggested insufficient evidence to reject the hypothesis that there are no direct paths between variables). The goodness of fit was assessed by Bentler’s comparative fit index (CFI) and the goodness of fit index (GFI). The threshold was 0.90, which indicated a good fit [51]. As a test of close fit, the root means the square error of approximation (RMSEA) was used. The values below 0.08 confirmed a good fit for the model.

Statistica V. A 13.0 statistical package (Tibco, 2020, Statsoft Poland, Cracow, Poland) was used to calculate descriptive statistics and ANOVA tests with α = 0.05 set as the level of statistical significance.

## 3. Results

The mean calendar age in the experimental group of the girls was 16.12 ± 0.39 years, with a mean value of body height—164.89 ± 6.08 cm and body weight—56.07 ± 1.92 kg. Boys from the experimental group were aged 16.21 ± 0.31 years, with a body height of 176.47 ± 6.21 cm and a body weight of 64.06 ± 11.89 kg. The control group girls were 16.12 ± 0.46 years old, with a body height of 163.92 ± 6.96 cm and a body weight of 57.27 ± 12.23 kg. Boys from the control group were 16.28 ± 0.38 years old, with body height—of 177.13 ± 5.98 cm and body weight—of 65.69 ± 10.89 kg.

Descriptive statistics of the baseline values of the biological age (predicted age at peak height velocity and maturity offset), anthropometric measurements (body height and body weight), body composition (body fat mass and body fat mass index), and fitness index, as well as changes between postintervention and baseline values in FI (Δ), are presented in Table 1.

The mean age at the peak of height velocity (APHV) was lower in girls than boys but was very similar between the EG and CG inside sex groups. A girl’s earlier maturation was statistically significant (F = 214.16, *p* < 0.001). Parallelly, the maturity offset (MO) in girls was about 3.5 years, while in boys—2.4 years. Differences were statistically significant (F = 139.83, *p* < 0.001). 

Detailed analyses of the anthropometric measurements, body composition, and cardiorespiratory fitness concerning HIIT effects and body fat status have been published elsewhere [10,42,43]. Briefly, the basic anthropometric features’ status was similar in experimental and control groups, and differences were not statistically significant (height: F = 0.02, *p* = 894, weight: F = 0.55, *p* = 0.46). However, some features were dependent on sex. Boys were taller and heavier. Body fat mass index (which indicated proportions of the fat mass concerning body height) was higher in girls than boys, but values of the FMI were very close in the experimental and control groups within the same-sex groups (F = 0.39, *p* = 532). Experimental groups did not significantly differ from control groups in preintervention values of the FI (F = 0.07, *p* = 0.791). The changes in FI were positive in all groups but much greater in experimental groups (both sexes). The greatest changes were observed in EG boys (mean = 3.01 pts), next in EG girls (mean = 1.88 pts), and in CG groups (boys and girls) they were very low and similar (circa 0.35 pts). Such differences were highly statistically significant between EG and CG (F = 10.21, *p* = 0.002). 

The full model (males and females) was analyzed in the first step of the analysis. Figure 1 shows the structural model and *β*-standardized coefficients for all its components. The model showed adequate fit: *β*^2^ = 1.25, *p* = 0.264, CFI = 0.993, GFI = 0.999 and RMSEA = 0.042 (0.000–0.234 95% CI) and *p* = 0.343. 

Table 1 shows the results of the path analysis for the whole group (sex as one of the exogenous factors). Age at peak height velocity had a significant negative effect on the level of changes (ΔFI) induced by the HIIT program (*β* = −0.270, *p* = 0.035). High-intensity interval training significantly positively affected changes in FI (*β* = 0.246, *p* = 0.002). Sex also had a significant direct positive effect on the level of physical efficiency (*β* = 0.344, *p* = 0.011). Statistically significant results suggested the moderation role of the sex and possible differences in model paths between males and females, which was verified in the second step of the analysis. In the model for the whole group of participants (without separation sex), the direct influence of the preintervention FMI on ΔFI was not observed (*β* = 0.107, *p* = 0.244).

Table 2 presents the direct and indirect effects of APHV, HIIT, sex, and preintervention FMI on ΔFI. The status of body fat mass (relative to body height) was negatively but non-significantly affected by APHV (*β* = −0.087, *p* = 0.459). The biological age range did not indicate the relationship between APHV and FMI. Thus, sex significantly negatively affected FMI values (*β* = 0.425, *p* < 0.001). It meant a higher level of FMI in girls than in boys, which was expected. 

Comparison of the *β*-standardized coefficients allowed for classifying exogenous variables affecting ΔFI in strength order. The highest coefficient was noted for sex, followed by the HIIT program and APHV (significantly influencing cardiorespiratory fitness effects). Indirect effects were trivial and possible to omit. 

Neither the APHV on ΔFI through preintervention FMI nor indirect influences of the sex on ΔFI through preintervention FMI was observed (*β* = −0.009, *p* = 0.532, *β* = −0.045, *p* = 0.268, respectively). 

**Figure 2:**
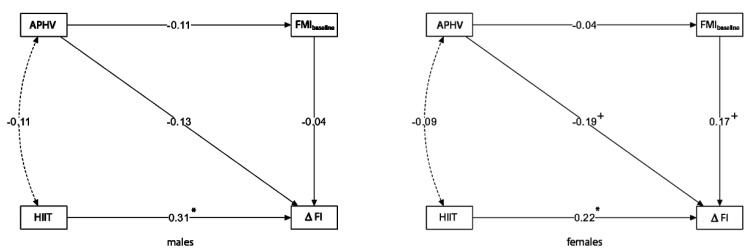
Path models, constructed for males and females, show the association between changes in FI and APHV, HIIT intervention, and sex as exogenous variables with the preintervention FMI. Abbreviations: APHV—age at peak height velocity, HIIT—HIIT groups (EG−1 coded, CG-0 coded), FMI_baseline_—fat mass index preintervention, ΔFI—changes in fitness index, *—statistically significant path, ^+^ *p* < 0.1 (very close to significance).

The second analysis step compared two models for males and females separately. Figure 2 shows structural models and *β*-standardized coefficients for all its components in both models. Both models showed adequate fit: boys—*β*^2^ = 0.113, *p* = 0.737, CFI = 0.999, GFI = 0.999 and RMSEA = 0.000 (0.000–0.260 95% CI) and *p* = 0.753; girls—*β*^2^ = 0.299, *p* = 0.084, CFI = 0.782, GFI = 0.999 and RMSEA = 0.150 (0.000–0.360 95% CI) and *p* = 0.118. Thus, the model for boys was slightly better fitted. 

Table 3 shows the results of the path analysis for boys and girls. Age at peak height velocity was very close to the significant negative direct effect on the level of changes induced by the HIIT program in girls (*β* = −0.193, *p* = 0.053) but not in boys (*β* = −0.135, *p* = 0.311). At the same time, the HIIT program had a significant positive direct effect on changes in FI in both sexes (boys: *β* = 0.313, *p* = 0.018; girls: *β* = 0.223, *p* = 0.026). In models for boys and girls, the direct influence of the preintervention fat level (FMI) on ΔFI was not observed in boys (*β* = −0.004, *p* = 0.742) and was very close to significance in girls (*β* = 0.174, *p* = 0.091). The status of body fat mass (relative to body height) was not directly affected by APHV in boys (*β* = −0.110, *p* = 0.430) or girls (*β* = −0.038, *p* = 0.728). Comparing *β*-standardized coefficients allowed for classifying exogenous variables affecting ΔFI in strength order. Both sexes observed the highest direct effect for the HIIT program and the next for APHV. Indirect influences of HIIT on ΔFI through preintervention FMI were observed neither for boys (*β* = 0.005, *p* = 0.761) nor girls (*β* = 0.007, *p* = 0.734). 

## 4. Discussion

This work aimed to assess the role of the biological age (APHV) in the relationship between preintervention fat mass index (FMI) and HIIT intervention in cardiorespiratory fitness (CRF) in adolescent boys and girls—concerning the moderation role of sex. The findings showed significant relations between all three factors (APHV, HIIT, and Sex) and changes in CRF after the intervention. In detail, earlier maturated adolescents gained greater CRF changes than later maturated peers. Differences between post- and preintervention CRF were statistically significantly greater in experimental groups than in control groups. Males received significantly greater changes compared with females. All factors affected ΔFI directly, and indirect relations were not observed. There was no significant relationship between baseline FMI level and ΔFI in the model for the entire group. However, FMI_baseline_ was significantly affected by APHV. Once the moderated role of the sex factor was confirmed, the models for both sexes were constructed separately. The HIIT program greatly, positively, and directly influenced CRF. Results were greater in boys. The APHV of the girls affected ΔFI very close to significance (*p* = 0.053), but such a relationship was not observed in boys (*p* = 0.311). In addition, in girls, changes in CRF were positive and very close to significance (*p* < 0.100) affected by FMI_baseline_. At the same time, boys had no similar relationship (*p* = 0.742). 

In the structural models (for the whole group and each sex), the indirect associations between exogenous and endogenous variables, initially hypothesized by the study, were not observed. The possible explanation could be that coefficient values of indirect effects in associations between APHV, Sex, and ΔFI mediated by the baseline FMI were too low and statistically insignificant. Studied factors did not affect baseline FMI level in such a way that could indirectly impact cardiorespiratory fitness. Further studies on indirect effects of the biological age, intervention programs, and the mediation role of the body composition in cardiorespiratory changes are needed.

This study showed the biological age’s direct, negative effect on cardiorespiratory changes. The role of biological maturation in HIIT effects was checked by Alvarez et al. [38]. This study showed the independent impact of HIIT on health parameters due to maturation. In contrast, our study showed the effect of biological age in girls but not boys. However, the study cited above [38] established maturation with Tanner stages. Biological age was a significant factor in differentiation between children and adolescents in morphology and functional state [2]. However, data are lacking to consider maturation status in HIIT effects. Therefore, further studies are needed. One of our previous studies [43] revealed a higher prevalence of HIIT positive effects in boys than girls. Due to observations in the relevant study differences in HIIT effects on CRF affected by APHV, there is a need to consider biological maturation as a factor decisive in introducing the HIIT program. It was observed that faster maturation is associated with the development of body morphology and physical performance [52]. Therefore, girls who were at higher APHV noted better improvement in CRF. This effect was not observed in boys. However, there is a need to remember that males are more susceptible to react positively to training loads than women [53]. However, our study sample was young adolescents before or during maturation, and therefore other factors may affect this observation, e.g., level of physical fitness [54].

High-intensity intervention training directly and positively affected cardiorespiratory fitness. Differences between post- and preintervention CRF were statistically significantly greater in experimental groups than in control groups. HIIT improves endurance and decreases BMI and body fat percentage [36]. Our previous study [10] showed a bodyweight and body fat decrease after HIIT, which also affected aerobic capacity. The other studies provide similar observations. It was reported by Bogataj et al. [55] that in obese girls, HIIT improves body composition and physical fitness simultaneously. A reduction in waist and abdominal circumference was also observed directly (associated with body fat) after HIIT intervention [56]. The abovementioned observations are supported by Tjonna et al. [57], who provided similar observations of HIIT effects but independently of sex. 

Physical education lessons provide a relevant setting to increase physical activity levels in adolescents [58]. Nowadays, it is essential to engage children and adolescents to participate in various forms of physical effort after the COVID-19 pandemic, during which physical activity levels decreased significantly [59,60]. However, introducing PE lessons to high-intensity exercises benefits students more [10,61]. The huge advantage of HIIT is the short duration time that fits a 45-min single PE lesson [8,10]. Moreover, it was proved that HIIT programs achieved broader health benefits than low- and moderate-intensity efforts [62]. It was observed that HIIT greatly influences body morphology and the cardiovascular system [31].

It was also shown by [63] Batacan et al. that muscle mass, strength, and strength with joint improvement increased in cardiovascular parameters. The meta-analysis conducted by Solera-Martinez et al. [34] confirmed the high effectiveness of different interval programs (HIIT with Tabata protocol) on metabolic parameters and cardiorespiratory fitness. Our study revealed these effects, in which boys and girls improved their CRF. To the abovementioned observation, a confirmation study by Hay et al. [64] observed improvement in CRF with a simultaneous decrease in body fat in 6 months. Brown et al. [65] confirmed that HIIT results in CFR improving among females.

Although this study showed positive changes in CRF after the HIIT intervention program in both sexes, boys received greater changes compared to females. Similar results were obtained by Engel et al. [40], who found improvements in aerobic performance variables in athletes, mainly boys aged 8–18, following a HIIT training protocol. Cvetković et al. [45] observed a significant improvement in physical fitness from 11- to 13-year-old boys and girls in a 12-week HIIT training program. Furthermore, in 14-year-olds in PE classes, Racil et al. [62] found greater improvement in maximal oxygen uptake and maximal aerobic speed following 12 weeks of HIIT or moderate-intensity interval training programs in boys and less in girls. Additionally, Camacho-Cardenosa et al. [66] compared changes in 11-year-olds following an HIIT program and found increased cardiorespiratory fitness in the experimental groups for both sexes. Therefore, the present study confirmed the importance of HIIT protocols in physical activity to improve cardiorespiratory fitness for boys and girls. 

Body fat mass at baseline was shown to have a positive direct effect on CRF in girls. Associations were very close to significance, while boys had no significant relationship. The positive coefficient value suggested that the higher level of body fat at baseline corresponded with the lower effectiveness of the intervention program in CRF. Positive changes were lower in adolescent girls with greater fat mass than slim peers. Such a mechanism does not seem to matter in boys. 

Those findings align with Bonney et al. [67] results, which indicated that increased body mass is associated with lower CRF. These results are also confirmed by Hsieh et al. [68] observations. Body mass is strongly correlated with cardiorespiratory fitness. It was also proved that increased fat mass is associated with decreased physical fitness in maturing girls [69]. However, in terms of HIIT effects, Ouerghi et al. [70] showed that obese subjects might benefit more from HIIT intervention in CRF and body composition than normal-weight ones.

Similarly, Lambrick et al. [71] reported results in which obese and normal-weight subjects improved their CRF level, but boys and girls were mixed in this study. On the contrary, another study [4] suggested that individuals, even in the average body mass index range but very close to the upper limit of the norm (close to overweight), did not achieve significant physical efficiency after the HIIT program. Our findings are in line with the results of Cvetkowić et al. [45] and Racil et al. [62]. The obtained results underline an unclear problem: is the HIIT intervention is effective for body fat reduction in relation to sex and what is the role of the baseline body composition? 

In our study, APHV impacted CRF, which suggested the significant role of maturation in CRF changes after HIIT. It was observed that advanced maturity status with a low CRF level is associated with obesity/overweight risk [72]. However, according to Mota et al. [73], maturation status seemed not decisive in CRF status, confirming our observation.

We are aware that our study has limitations. One is APHV, calculated from a formula containing only body height and weight (not sitting height). We do not monitor daily activity and nutrition. Although the number of participants was sufficient, the disproportion between boys and girls and between experimental and control groups could have affected the results. Another limitation was bias in sex distribution, and only one secondary school was involved in the study. We do not continuously monitor heart rate, and cardiorespiratory fitness was assessed through a fitness index acquired from the Harvard Step Test, which was less estimated than other field tests.

On the other hand, this leads to some strengths. There is a need to highlight that the Harvard Step Test is more often used in PE lessons than other field tests assessing CRF. Our study was conducted in natural school settings where the possibility of using a heart rate monitor is very uncommon; therefore, we show a cheap and effective alternative. Moreover, our study participants were of the same age and education level. It also needs to emphasize the statistic methodology, which reveals a broad association structure between analyzed factors considering HIIT and health markers.

## 5. Conclusions

This study sought to go beyond the well-known and widely described assessment of the simple and direct relationships between variables. This study confirmed the role of APHV in girls but not boys. It meant that the adolescent boys could conduct the same HIIT program, while the HIIT program should be tailored to biological maturity in girls. Physiological mechanisms determining the volume of changes in cardiorespiratory fitness depend on APHV and baseline fat mass differently and with different strengths in both sexes. There was a sex moderation effect. When the effects of HIIT and APHV were analyzed according to the sexes, the significant influence of the APHV on ΔFI was observed (very close to significant, in fact) in girls. The variables used in this study affected CRF directly with no indirect influence through the baseline FMI. There were no moderation effects of the FMI_baseline_ in the relationship between APHV, HIIT, and sex on CRF changes. Body composition did not play an indirect role in such associations.

## Figures and Tables

**Table 1:** Biological age, anthropometric measurements, body composition, and fitness index at baseline statistics.

Factor	Boys		Girls	
	Experimental	Control	Experimental	Control
	Mean (±sd) 95% CI	Mean (±sd) 95% CI	Mean (±sd) 95% CI	Mean (±sd) 95% CI
APHV	13.81 (0.46) 13.64–13.98	13.91 (0.41) 13.72–14.09	12.58 (0.50) 12.43–12.74	12.67 (0.50) 12.52–12.81
MO	2.40 (0.40) 2.25–2.55	2.37 (0.53) 2.13–2.61	3.54 (0.57) 3.37–3.72	3.45 (0.58) 3.28–3.62
Body Height	176.47 (6.21) 174.19–178.74	177.13 (5.98) 174.41–179.86	164.89 (6.08) 163.00–166.79	163.92 (6.96) 161.88–165.97
Body Weight	64.06 (11.89) 59.70–68.42	65.69 (10.89) 60.74–70.65	56.08 (7.48) 53.75–58.41	57.27 (12.23) 53.68–60.86
FMI	3.51 (2.44) 2.62–4.41	3.24 (2.10) 2.29–4.20	5.55 (1.39) 5.11–5.98	6.30 (2.60) 5.54–7.06
FI	44.59 (3.50) 43.30–45.87	43.79 (3.32) 42.28–45.30	43.43 (4.62) 41.99–44.87	44.60 (3.96) 43.44–45.76
ΔFI	3.01 (4.22) 1.46–4.56	0.36 (3.25) −1.12–1.84	1.88 (4.04) 0.62–3.14	0.34 (3.19) −0.59–1.28

APHV—the age at peak height velocity, MO—maturity offset, FMI—fat mass index, FI—physical fitness index, ΔFI—delta: the difference between posttest–pretest (baseline) results.

**Table 2:** Direct and indirect effects of APHV, HIIT, sex, preintervention FMI on ΔFI.

95% Confidence Intervals								
DV	Predictor	Estimate	SE	Lower	Upper	*β*	z	*p*
direct effect								
ΔFI	APHV	−1.361	0.644	−2.623	−0.098	−0.270	−2.112	0.035
ΔFI	HIIT	1.878	0.614	0.675	3.080	0.246	3.060	0.002
ΔFI	Sex	2.722	1.066	0.633	4.812	0.344	2.553	0.011
ΔFI	FMI_baseline_	0.165	0.142	−0.113	0.443	0.107	1.164	0.244
FMI_baseline_	APHV	−0.287	0.387	−1.046	0.472	−0.087	−0.741	0.459
FMI_baseline_	Sex	−2.188	0.607	−3.378	−0.998	−0.425	−3.604	<0.001
indirect effect								
APHV ⇒ FMI_baseline_ ⇒ ΔFI		−0.047	0.076	−0.196	0.101	−0.009	−0.625	0.532
Sex ⇒ FMI_baseline_ ⇒ ΔFI		−0.361	0.326	−1.000	0.278	−0.046	−1.108	0.268

**Table 3:** Direct and indirect effects of APHV, HIIT, preintervention FMI on ΔFI in boys and girls.

95% Confidence Intervals								
DV	Predictor	Estimate	SE	Lower	Upper	*β*	z	*p*
boys								
direct effect								
ΔFI	APHV	−1.233	1.216	−3.617	1.151	−0.1345	−1.014	0.311
ΔFI	HIIT	2.552	1.075	0.444	4.660	0.3126	2.373	0.018
ΔFI	FMI_baseline_	−0.077	0.234	−0.535	0.381	−0.0435	−0.330	0.742
FMI_baseline_	APHV	−0.570	0.722	−1.986	0.846	−0.1099	−0.789	0.430
indirect effect								
HIIT ⇒ FMI_baseline_ ⇒ ΔFI		0.044	0.144	−0.239	0.327	0.005	0.304	0.761
girls								
direct effect								
ΔFI	APHV	−1.443	0.746	−2.905	0.019	−0.193	−1.934	0.053
ΔFI	HIIT	1.644	0.737	0.201	3.088	0.223	2.232	0.026
ΔFI	FMI_baseline_	0.312	0.184	−0.050	0.673	0.174	1.690	0.091
FMI_baseline_	APHV	−0.157	0.452	−1.043	0.729	−0.038	−0.347	0.728
indirect effect								
HIIT ⇒ FMI_baseline_ ⇒ΔFI		−0.049	0.144	−0.331	0.233	−0.007	−0.340	0.734

## Data Availability

Data are available upon request due to ethical restrictions regarding participant privacy. Requests for the data may be sent to the corresponding author.
